# Comprehensive Analysis of DNA Methylation and Transcriptome to Identify PD-1-Negative Prognostic Methylated Signature in Endometrial Carcinoma

**DOI:** 10.1155/2022/3085289

**Published:** 2022-05-18

**Authors:** Lu Cao, Xiaoqian Ma, Pengfei Rong, Juan Zhang, Min Yang, Wei Wang

**Affiliations:** ^1^Department of Radiology, The Third Xiangya Hospital, Central South University, 138 Tongzipo Road, Yuelu District, Changsha, China; ^2^The Institute for Cell Transplantation and Gene Therapy, The Third Xiangya Hospital, Central South University, 138 Tongzipo Road, Yuelu District, Changsha, China

## Abstract

**Background:**

Epigenetic mechanism plays an important role in endometrial carcinoma (EC). This study was designed to analyze the epigenetic mechanism between DNA methylation-driven genes (DEDGs) and drugs targeting DEDGs and to develop a DEDG score model for predicting the prognosis of EC.

**Methods:**

Expression profile and methylation profile data of PD-1-negative EC samples were obtained from TCGA. To obtain intersected DEDGs, differentially expressed genes (DEGs) and differentially methylated genes from tumor tissues and normal tissues were analyzed by limma. A linear discriminant classification model was constructed using the gene expression profile of DMDGs, methylation profile of TSS1500, TSS200, and gene body regions. Principal component analysis (PCA) and ROC analysis were conducted. The protein-drug interactions analysis of DMDGs was performed using Network Analyst 3.0 tool. Lasso Cox regression analysis was used to screen prognostic DNA methylation driving gene and to build a risk score model. The ROC curve and Kaplan-Meier survival curve were plotted to evaluate the model prediction capability.

**Results:**

A total of 96 DMDGs were screened from the three regions, distributed on 22 chromosomes, with consistent methylation patterns in different gene regions. Both the expression profile and methylation profile of the three regions can neatly distinguish tumor samples from normal ones, with a high classifying performance. A gene signature, which consisted of ELFN1-AS1 and ZNF132, could classify EC patients into a high-risk group and low-risk group. Prognosis of the high-risk group was significantly worse than that of the low-risk group. The risk model showed a high performance in predicting the prognosis of EC.

**Conclusion:**

We successfully established a risk score system with two DMDGs, which showed a high prediction accuracy of EC prognosis.

## 1. Introduction

Endometrial carcinoma (EC) is the fourth leading malignant tumor among women in 2021, with more than 410000 new cases being diagnosed in 2020 [1, 2]. EC usually occurs in postmenopausal women, with an average onset age of 68 years old [3]. Most endometrial cancers were diagnosed at the local stage and mostly can be cured by taking surgery, with a 5-year survival rate of about 95% [4]. Patients with advanced and recurrent EC face great treatment challenges, and the 5-year overall survival (OS) rate of patients with distant diseases is only 17% [5]. Therefore, it is necessary to accurately diagnose EC and improve the treatment of EC. An ongoing study demonstrated that predictive biomarkers (e.g., p53 or L1 cell adhesion molecule L1CAM) may have a promising effect on improving the clinical outcome of EC [6].

Cancer development is usually driven by genetic and epigenetic changes and complex crosstalk between many different pathways [7]. These changes involve DNA methylation, histone modifiers and readers, chromatin remodelers, microRNAs, and other components of chromatin [8]. As an important epigenetic mechanism, abnormal DNA methylation is considered a biomarker for early diagnosis and prognostic monitoring of many cancers [9]. Short stature homeobox gene two (SHOX2), RAS association domain family 1A (RASSF1A), and prostaglandin E receptor 4 gene (PTGER4) methylation has been reported to be closely related to lung cancer diagnosis and prognosis [10]. According to the results of a liquid biopsy, Hassan et al. indicated that SALL1, WNT5 *α*, LRP1, and CDH2 have diagnostic significance for children with acute lymphocytic leukemia [11]. In high-grade serous ovarian cancer, hypermethylated NCALD and LAMA3 are associated with prognosis and chemotherapy resistance in advanced patients [12]. In addition, a number of studies have identified panels of multiple methylation driving genes to predict the prognosis of colorectal cancer [13], gastric cancer [14], and hepatocellular carcinoma [15]. However, there is a lack of a comprehensive panel of methylation markers for EC [16].

Many previous studies have shown that new gene markers are helpful to tumor prognosis and diagnostic evaluation. Previously, Lai et al. identified 9 new tumor markers [17] based on proteomic data using the LASSO method. These proteins can predict the response to immunotherapy, chemotherapy, and targeted therapy. Liu et al. [18] filtered the characteristics of focal death-related 6 lncRNAs using the method of comprehensive bioinformatics. Tan et al. [19] screened 11 genes related to lipid metabolism based on the gene set associated with lipid metabolism, which can be used as prognostic markers of endometrial cancer to predict the response to chemotherapy and immunotherapy. Ruan et al. [20] used bioinformatics methods to identify the characteristics of novel epithelial mesenchymal transformation-related genes for the prognosis of endometrial cancer. These are mainly results achieved through gene expression profiling. They focus on all patients and specific gene concentration and pay limited attention to different types of patients, especially patients with PD-1 negative.

In this study, we systematically analyzed the expression profile data of PD-1-negative EC samples from The Cancer Genome Atlas (TCGA, https://www.tumorfusions.org/) and DNA methylation data to screen DMDGs and predicted their targeting drugs. Further analysis on DMDGs developed a risk signature that can monitor the prognosis of EC, contributing to a better clinical management of EC.

## 2. Materials and Methods

### 2.1. Sample and Data of EC

Tissue expression data, methylation data based on Illumina Human Methylation 450BeadChip array, and clinical follow-up information of EC samples were all downloaded from TCGA. After excluding PD-1-positive samples (the expression of PD-1 in tumor tissues was higher than the mean value of PD-1 expression in normal tissues), a total of 208 primary tumor samples and 35 normal samples were obtained. Methylation data were obtained from 163 tumor samples and 46 normal samples.

Differential expression analysis and differential methylation analysis.

DEGs between tumor tissues and normal tissues were analyzed by R package limma [21], and the *P* value was adjusted by the Benjamini and Hochberg (BH) [22] method. limma was also used to analyze differential methylation of tumor tissues and normal tissues, and differential methylation CpG sites were determined with FDR < 0.05 and absolute delta *β* (original methylation intensity of each probe) value > 0.3 as the thresholds. The CpG locus and gene matching file came from the Illumina website (https://www.illumina.com/).The average *β* values were calculated from the region covering -200 to -1500 nucleotides upstream of Transcription Start Site (TSS1500) from Transcription Start Site to -200 nucleotides upstream of Transcription Start Site (TSS200) and gene body. The differential methylation CpG sites were calculated by limma; here, *FDR* < 0.01 and delta *β* values > 0.3 were defined as hypermethylated regions, while those with FDR < 0.01 and delta *β* values < -0.3 were considered as demethylated regions.

### 2.2. Functional Enrichment Analysis

ClusterProfiler [23] database was used to perform gene ontology (GO) enrichment analysis and Kyoto Encyclopedia of Genes and Genomes (KEGG) path annotation analysis of DEGs and DMGs. *P* < 0.05 was set as the threshold for filtering significant functional events.

### 2.3. Identification of DMDGs

DNA methylation data and differentially expressed mRNA data were integrated to determine the intersection of DEGs and differentially methylated genes by Venn diagram. The concentrated genes were DMEGs, which were further divided into four groups, namely, HypoUp, HypoDown, HyperUp, and HyperDown. The grouping criteria were shown in Table 1.

### 2.4. Performance Evaluation of Class Energy Estimation Based on DMDGs

A linear discriminant classification model was developed using the gene expression profile of DMDGs. Methylation profile of gene body, the methylation spectrum of TSS1500 region and the methylation spectrum of TS200 region, and PCA (principal component analysis) analysis and ROC analysis were conducted. At the same time, leave-one-out cross-validation (LOOCV) [24] method was applied to verify the effect of classification.

### 2.5. Identification of Potential Target Therapeutic Drugs

The DrugBank [25] database was used to identify drugs targeting upregulated DMDG. NetworkAnalyst 3.0 [26] (http://www.networkanalyst.ca/) is a visual analysis web platform supporting comprehensive analysis and interpretation of system-level gene expression data. In this paper, the study of the protein-drug interaction on DMDGs was carried out using NetworkAnalyst 3.0 tool, and the network map of gene-drug interaction was generated.

### 2.6. Construction of Prognosis Scoring Model Based on Postestablishment Classification of DMDGs

PD-1-negative samples were randomly divided into training set (*n* = 104) and verification set (*n* = 104). According to the expression of DMEGs and survival data, LASSO regression analysis with 1000 repetition was performed in the training set, and tenfold cross-validation was used to develop a prognosis score model. The risk score of the samples was calculated and grouped by the model. We further examined the accuracy of the model using Kaplan-Meier (KM) analysis and receiver operating characteristic (ROC) curve analysis in the internal and external verification sets.

### 2.7. Statistical Analysis

The statistical analysis of all the data in this study were carried out in R software. *P* < 0.05 as the cut-off value was considered to be statistically significant.

## 3. Results

### 3.1. Determination and Functional Analysis of DEGs

**Figure 1:**
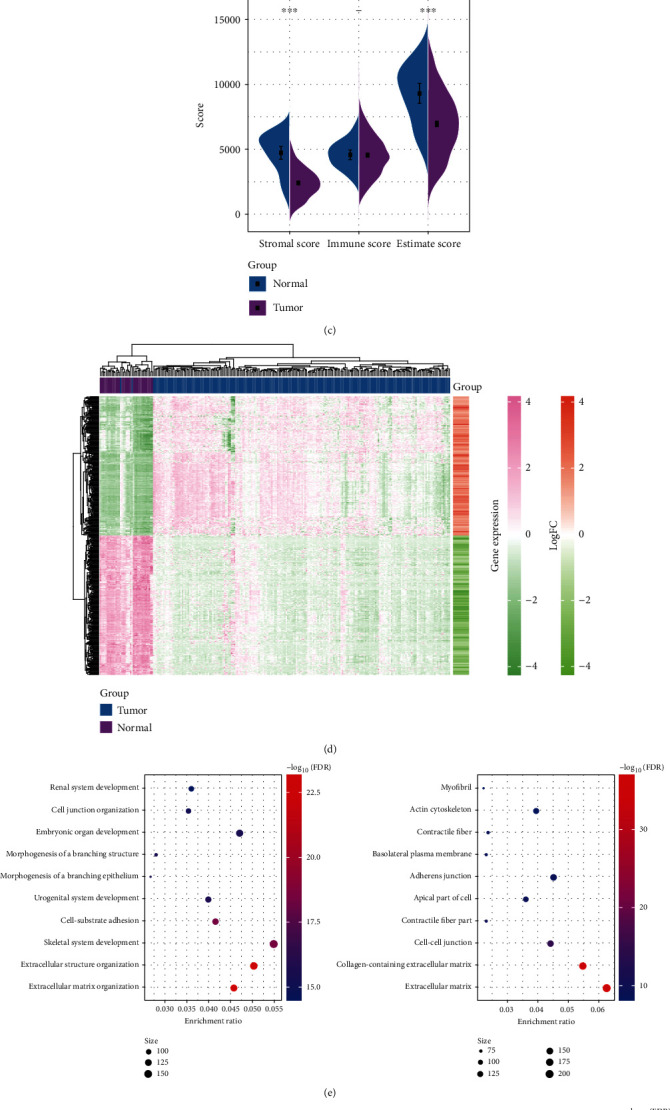
Determination and functional analysis of differentially expressed genes. (a) The difference of PD-1 expression between EC and normal tissues. (b) Volcanic map of DEGs between tumor tissue and normal tissue. (c) The difference of stromal score and immune score and ESTIMA TE score between tumor tissue and normal tissue. (d) Unsupervised hierarchical clustering heat map of differential genes. (e) Top 10 GO analysis of 4153 DEGs from the aspects of BP, CC and MF. (f) Top 10 KEGG analysis of 4153 DEGs.

The difference analysis of PD-1 expression between tumor tissue and normal tissue showed that the expression of PD-1 in tumor tissue was significantly up-regulated (Figure 1(a)). According to the screening criteria of FDR < 0.01 and |log2FC| > 1, 4153 DEGs incorporating 1309 upregulated DEGs and 2844 downregulated DEGs were detected from 208 EC tissues and 35 normal tissues (Figure 1(b)). Between tumor tissue and normal tissue, there were significant differences in immune microenvironment scores, including matrix score and ESTIMATE score. These two scores in tumor tissue were significantly lower than those in the normal tissue (Figure 1(c)). Unsupervised hierarchical cluster analysis on 4153 DEGs demonstrated that DEGs could clearly distinguish tumor samples from normal samples (Figure 1(d)). Then, from the results of GO and KEGG enrichment analyses of differential genes, it could be found that 4153 differential genes were enriched to 107 pathways, 1582 biological process (BP), 134 cellular component (CC), and 80 molecular functions (MF). The most significantly enriched BP included renal system development, cell junction organization, and embryonic organ development. The CC showing the most significant correlation with 4153 differential genes were actin cytoskeleton, adherens junction, and cell-cell junction, and the most significantly enriched MF of these genes were cell adhesion molecule binding, DNA-binding transcription activator activity, actin binding and so on (Figure 1(e)). Furthermore, the results of KEGG demonstrated that the differential genes were mainly involved in the regulation of PI3K-Akt signaling pathway, MAPK signaling pathway, and focal adhesion and so on (Figure 1(f)).

### 3.2. Screening and Functional Analysis of Differentially Methylated Genes

**Figure 2:**
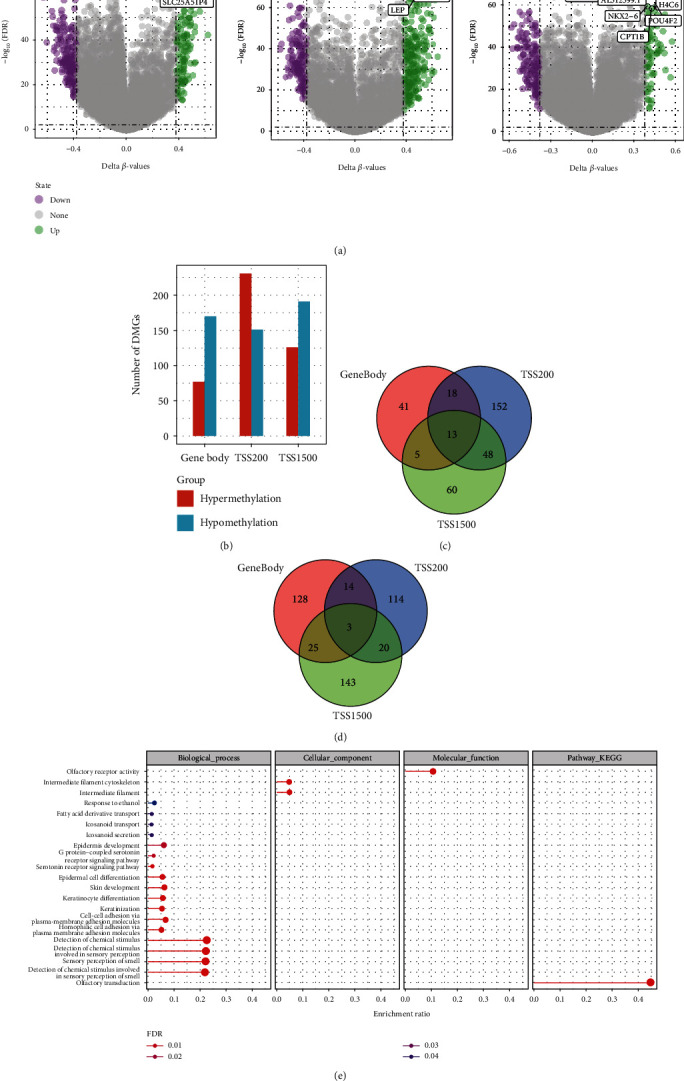
Screening and functional enrichment analysis of differential methylation genes. (a) Volcano map of differentially methylated genes in TSS1500, TSS200, and gene body regions. (b) Histogram of hypermethylation and demethylation genes in TSS1500, TSS200, and gene body regions. (c) Overlap analysis of hypermethylation genes in TSS1500, TSS200, and gene body regions. (d) Venn diagram of demethylated genes in TSS1500, TSS200, and gene body regions. (e) Differential methylation genes KEGG and GO enrichment bubble maps, in which the color from blue to red represented FDR from large to small, and dot size represented enrichment to the number of genes; the larger the dot, the more genes were enriched.

In view of the effect of methylation on gene expression, we analyzed the methylation data of 163 tumor samples and 46 normal samples. A total of 1063 differentially methylated genes were identified from TSS1500, TSS200, and gene body regions. Specifically, the TSS1500 region contained 126 hypermethylation genes and 191 demethylation genes; the TSS200 region contained 231 hypermethylated genes and 151 demethylated genes; gene body region contained 77 hypermethylation genes and 170 demethylation genes (Figure 2(a)). After comparing the proportion of differential hypermethylation and differential demethylation genes in different regions, we found that the number of hypermethylation genes in gene body and TSS1500 regions was significantly fewer than that of demethylation genes. In the TS200 region, the number of hypermethylated genes was higher than that of demethylated genes (Figure 2(b)). Overlap analysis on the hypermethylation genes in the three regions indicated that 13 genes were present in all the three regions (Figure 2(c)). Similarly, the Venn diagram of the intersection of demethylated genes showed the presence of the three genes in all the three regions (Figure 2(d)). Furthermore, GO and KEGG enrichment analyses were conducted to define the most significantly enriched GO terms and pathways of the differential methylation genes. Here, the data revealed that the differential methylation genes were mainly related to 17 BP (including epidermis development, cell-cell adhesion, and G protein-coupled serotonin receptor signaling pathway), 2 CC (intermediate filament cytoskeleton, intermediate filament cytoskeleton), 1 MF (olfactory receptor activity), and 1 KEGG pathway (olfactory transduction) (Figure 2(e)).

### 3.3. Identification of DMDGs and Biological Processes and Regulatory Mechanisms of DMDG-Related Substance Passing and Controlling

**Figure 3:**
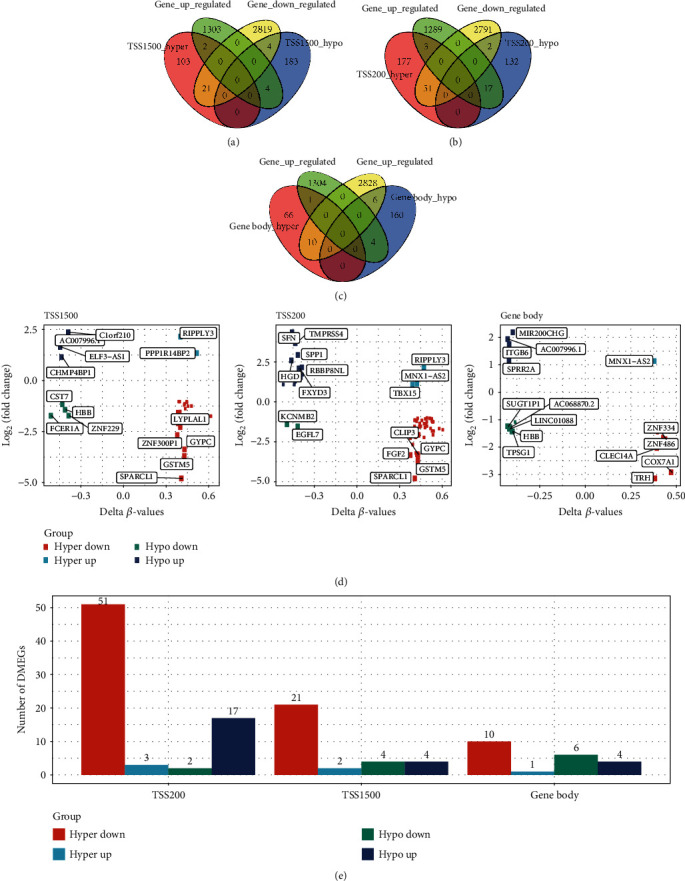
Identification of DMDGs. (a) Venn diagram of DEGs and differentially methylated genes in the TSS1500 region. (b) Venn diagram of DEGs and differentially methylated genes in TSS200 region. (c) Venn diagram of the intersection of DEGs and differential methylation genes in the gene body region. (d) Quadrant maps of DEGs and differentially methylated genes in TSS1500, TSS200, and gene body regions. (e) Histograms of four regulatory patterns of DEGs and differentially methylated genes in TSS1500, TSS200, and gene body regions.

**Figure 4:**
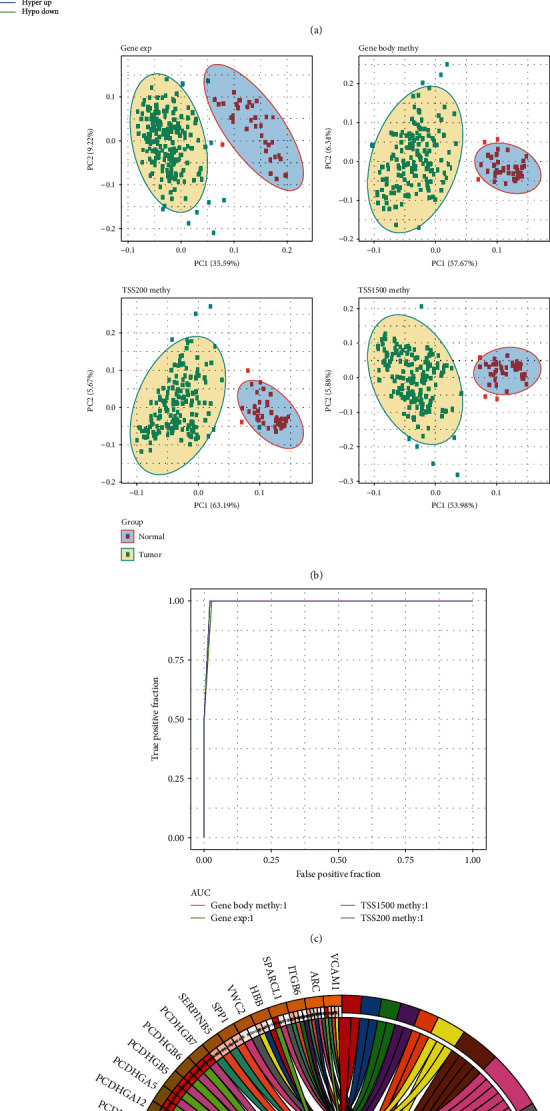
Chromosomal mapping and biological processes and mechanisms of DMDGs. (a) The location and distribution of DMDGs on chromosomes. (b) Expression of DMDGs and PCA analysis of methylation. (c) The ROC curve of classification performance of linear discriminant model based on the DMDG expression profile and methylation profile. (d) Results of KEGG pathway and GO enrichment in DMDGs, different colors represented different pathways, and wiring indicated a relationship between genes and pathways.

Overlap analysis was performed on differential methylated genes and DEGs in TSS1500, TSS200, and gene body region, respectively, and the genes in the crossover were found to be DMDGs. There were 31 DMDGs in TSS1500, 73 DMDGs in TSS200, and 21 DMEGs in gene body (Figures 3(a)–3(c)).  Figure 3(d) showed the methylation multiple and differential expression multiple of these DMDGs, and some genes, such as GYPC, GSTM5, SPARCL1, and RIPPLY3, also appeared in different regions at the same time. Among the three regions, the proportion of DMDGs of HyperDown type was the highest (Figure 3(e)). By combining different types of DMDGs in the three regions, a total of 96 DMDGs were obtained: 23 belonged to HypoUp type, 4 belonged to HyperUp type, 10 belonged to HypoDown type, and 59 belonged to HyperDown type. The distribution of 96 DMDGs on the genome was analyzed. The results showed that they were distributed on 22 chromosomes, in which most of the DMDGs were on chr1, chr2, chr5, and chr19 chromosomes, and the methylation patterns of DMDGs in different gene regions were consistent (Figure 4(a)). To explore the difference between gene expression and DNA methylation data in tumor and normal samples, a linear discriminant classification model was developed based on gene expression profile of DMDGs and methylation profile of the three regions, respectively. According to the results of PCA analysis, both the expression profile and the methylation profile of the three regions can neatly classify tumor samples and normal samples (Figure 4(b)). ROC analysis showed that the expression profiles and methylation profiles of the three regions had ultrahigh classification performance **(**Figure 4(c)). Our study on biological processes and regulatory mechanisms of 96 DMDG enrichment indicated that 14 terms were enriched, including cell-cell adhesion via plasma-membrane adhesion molecules, homophilic cell adhesion via plasma membrane adhesion molecules, and protein complex involved in cell adhesion, which were all related to cell adhesion (Figure 4(d)).

### 3.4. Screening and Verification of Screening and Syndromes of Potential Therapeutic Compounds in DMDGs

**Figure 5:**
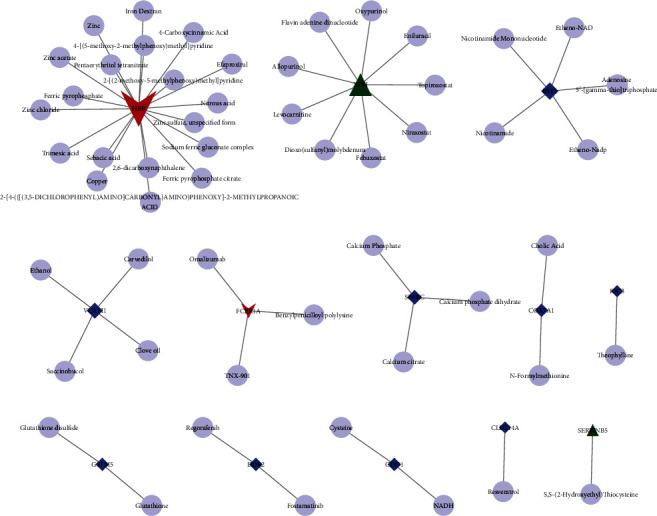
Interaction network between DMEGs and drug.

**Figure 6:**
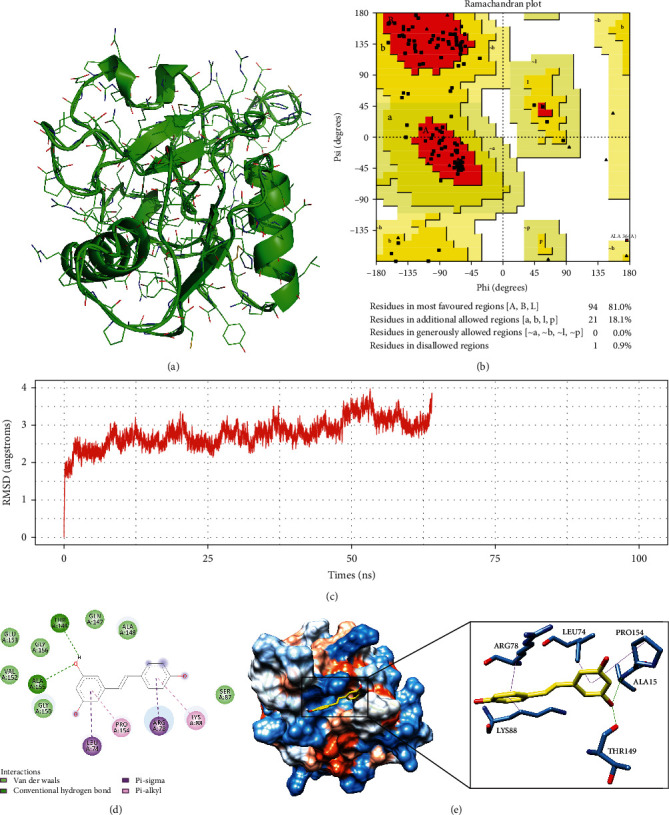
Molecular docking of resveratrol and CLEC14A. (a) 3D structure of CLEC14A protein predicted in SwissModel. (b) The Ramachandran plot of CLEC14A protein structure, and red, yellow, and white were the conformational permissible, permissible, and nonpermissible regions, respectively. (c) The change curve of the RMSD value of protein skeleton in the process of 100 ns molecular dynamics. (d) Two-dimensional interaction diagram of resveratrol and CLEC14A protein, the green dotted line represented the hydrogen bond, and the pink dotted line represented the hydrophobic interaction. (e) The 3-dimensional docking results of resveratrol and CLEC14A protein, the yellow structure represented the ligand, and the heteroatoms on it were shown as elements. The steel blue structure was an amino acid residue, and the heteroatoms on it were also shown as elements.

To identify drugs related to DMDGs, protein-chemical compound interaction analysis was carried out. Table 1 lists 13 DMDGs and compounds, including BST1, CDO1, CLEC14A, COX7A1, DDR2, FCER1A, GSTM5, HBB, RIC3, SERPINB5, SPARC, VCAM1, and XDH. The protein-chemical compound interaction network more intuitively displayed the interaction between DMDGs and drugs (Figure 5). Considering there was a lack of research on CLEC14A, molecular docking analysis was conducted by taking resveratrol-CLEC14A as an example. Firstly, the 3D structure of CLEC14A protein was predicted by SwissModel (https://swissmodel.expasy.org/). There were large neutral amino acid transporters in CLEC14A, and small subunits showed a high sequence similarity, which was an optimal model plate for modeling (Figure 6(a)). The results of Ramachandran plot analysis in PDBSum (http://www.ebi.ac.uk/thornton-srv/databases/cgi-bin/pdbsum) showed that 99.1% of the residues were in the allowable area, indicating a high accuracy of predicted structure (Figure 6(b)). Stability evaluation of the protein model using root-mean-square deviation (RMSD) method demonstrated that in the process of molecular dynamics simulation of 100 ns, the RMSD value of protein skeleton (protein-backbone) only slightly increased before 10 ns, and then, the whole protein remained in a relatively stable state, which confirmed the relative stability of CLEC14A protein in molecular dynamics simulation (Figure 6(c)). Furthermore, analysis on the docking mode between resveratrol and CLEC14A protein using Autodock Vina showed that resveratrol had a strong binding to CLEC14A protein mainly through hydrogen bond interaction with amino acid residues TYR149 and ALA155 in CLEC14A protein and hydrophobic interaction with amino acid residues such as PRO154, LEU74 ,ARG78, and LYS88 (Figure 6(d) and 6(e)).

### 3.5. Establishment of a Prognostic Model Based on DMDGs

**Figure 7:**
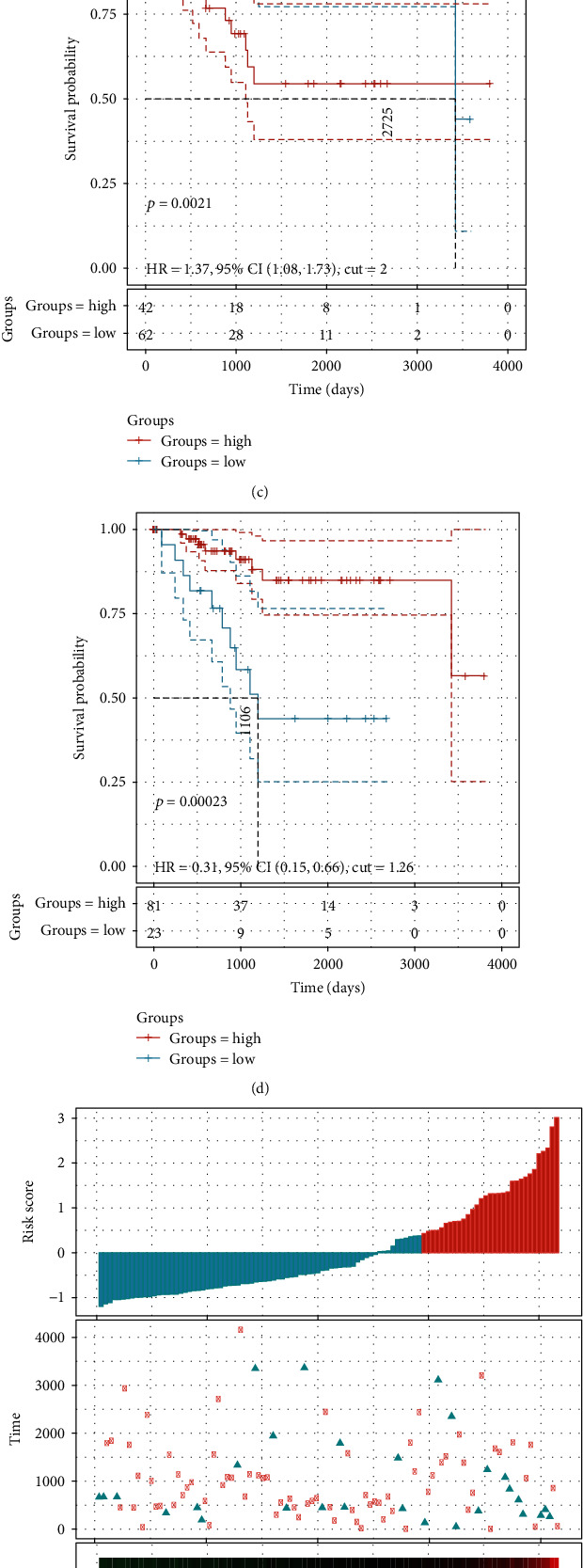
Prognostic model was established based on DMDGs. (a) The frequency of each gene combination in 1000 Lasso regression. (b) The variation coefficient locus and standard deviation distribution of different lambdas. (c) The Kaplan-Meier curve of the patients who were grouped according to the expression of ELFN1-AS1. (d) The Kaplan-Meier curve of patients who were grouped according to the expression of ZNF132. (e) The survival time, survival status, and 2-gene expression of risk score in the training set. (f) ROC curve of 2-gene signature classification. (g) Kaplan-Meier curve of samples with different risks in the training set.

To identify genes related to the prognosis of EC from DMDGs, the expression of 96 DMDGs and survival data were analyzed by LASSO regression analysis in the training set. According to the number of times each probe appeared during 1000 times, the combination with the highest frequency contained two genes, ELFN1-AS1 and ZNF132 **(**Figure 7(a)). The immune infiltration score of each patient was evaluated by R software package estimate. Through analyzing the relationship between these two genes and immune infiltration, it can be observed that they had a significant negative correlation with immune microenvironment infiltration (Supplementary figure 1). We further analyzed the variation coefficient trajectories of different lambdas and the distribution of standard deviation of models under different lambdas, and the risk score model was obtained: Risk score = 0.224∗ENSG00000236081 (ELFN1 − AS1) − 1.035∗ENSG00000131849 (ZNF132) (Figure 7(b), Table 2). After using the two genes in risk score system to divide patients into groups, we found that patients with low expression of ELFN1-AS1 showed a higher survival rate and those with high expression of ZNF132 had obvious survival advantage (Figures 7(c) and 7(d)). The risk score of each sample was calculated and arranged in the training set, and the results showed that the survival time of the sample with a high-risk score was significantly shorter than that with a low-risk score, suggesting that the sample with a high-risk score may have a worse prognosis (Figure 7(e)). ROC analysis of the prognostic classification of risk score demonstrated that the AUC values of 1-, 3-, and 5-year ROC curves were all higher above 0.7 (Figure 7(f)). The *z*-score cut-off value based on risk score was determined by R software package maxstat, and the samples were categorized into the high-risk group (*n* = 36) and low-risk group (*n* = 68). The survival outcome of patients in the high-risk group was found to be significantly worse than that in the low-risk group (Figure 7(g)).

### 3.6. Evaluation of Prognostic Model

**Figure 8:**
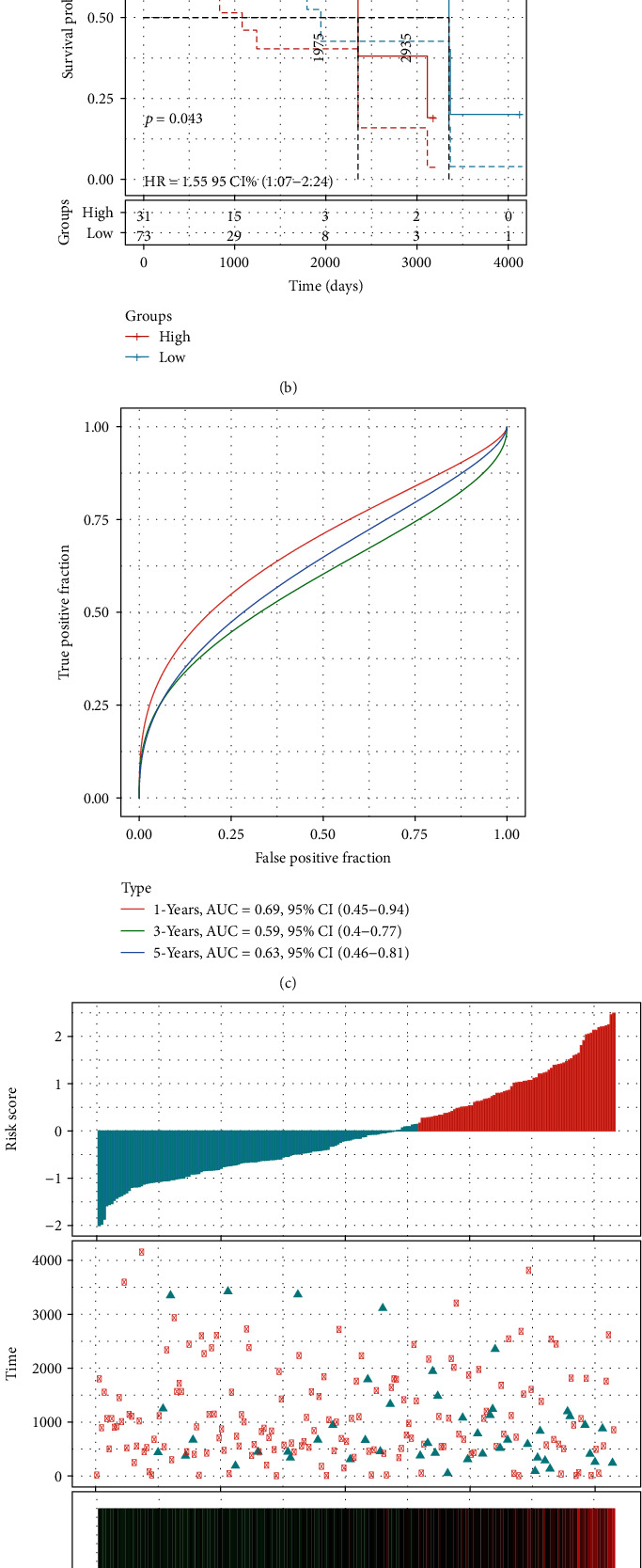
Verification of the 2-gene signature. (a) The risk score distribution, survival status scatter diagram, and expression heat map of 2 genes in the verification set. (b) Kaplan–Meier curves based on the log-rank test were created to visualize the prognostic value of 2-gene signature in the verification set. (c) The ROC curve over time in the validation set. (d) The risk score of the samples, distribution of survival time and state, and the expression heat map of ELFN1-AS1 and ZNF132 in the PD-1-negative cohort. (e) Kaplan–Meier curves based on the log-rank test were generated to visualize the prognostic value of the 2-gene signature in the entire PD-1-negative cohort. (f) ROC curve with time in PD-1-negative cohort.

The same method used in the training set was employed to evaluate the accuracy of the model in the verification set and the whole PD-1-negative cohort. Through the arrangement of the risk scores of the samples in each cohort, it could be observed that the number of survival patients gradually decreased with the increase of risk score whether in the verification set or the entire PD-1-negative cohort. The expression of ELFN1-AS1 was also upregulated with the increase of risk score, while ZNF132 showed an opposite trend (Figures 8(a) and 8(d)). The Kaplan-Meier curves based on the log-rank test were plotted to visualize the prognostic value of 2-gene signature in validation set and the entire PD-1-negative cohort. Consistent with the results of the verification set, high-risk samples showed a higher death risk than low-risk samples (Figures 8(b) and 8(e)). The ROC curves in the verification set and the entire PD-1-negative cohort demonstrated that the risk system performed well in predicting 1-year and 5-year survival rates of patients with EC, with an AUC higher than 0.6 (Figures 8(c) and 8(f)).

## 4. Discussion

At present, prediction of EC prognosis is still largely based on routine pathological indexes such as clinical stage, degree of tumor differentiation and invasion, and vascular infiltration. The development of molecular biology has enriched the knowledge of genetic mechanism of EC, contributing to the development of new methods for diagnosis, treatment, and prognosis [27]. Antiprogrammed cell death protein 1 immunotherapy has now been approved by the U.S. Food and Drug Administration for treating the female subgroup with metastatic EC [6]. The effective response to anti-PD-L1/anti-PD-1 therapy requires the establishment of a complete immune cycle, each step of which is regulated by epigenetic modification [28]. In view of such an effect of the two, the PD-1-positive EC samples from TCGA were eliminated, and DMDGs were screened according to the joint analysis of expression data and methylation data of the remaining EC samples.

Through the overlap analysis of differential methylation genes in DEGs, TSS1500, TSS200, and gene body regions, we obtained a total of 96 DMDGs from the three regions, which were distributed on 22 chromosomes, and the most abundant genes were found on chr1, chr2, chr5, and chr19 chromosomes. Additionally, a linear discriminant classification model was developed based on the gene expression profile of DMDGs and the methylation profile of the three regions, and the results of PCA analysis confirmed that both the expression profile and methylation profile of the three regions can clearly classify tumor and normal samples. The above 96 DMGMs were enriched in 14 terms, and the most obvious enrichment items were biological functions and mechanisms related to cell adhesion; therefore, DMGMs may be involved in regulating the pathological process of EC.

At the same time, the drug components targeting DMGMs were analyzed through the developing interactions network of protein-compounds, and it was found that the compounds targeting HBB were the most. Among several compounds targeting DMDGs, some have been shown to have potential anticancer effects. For example, Hirsh et al.'s study found that theophylline induces apoptosis of cancer cells, and they also mentioned an ongoing clinical trial using theophylline to treat patients with lung cancer [29]. Regorafenib is a multitarget kinase inhibitor approved for the treatment of metastatic colorectal cancer patients who are unresponsive to standard chemotherapy [30]. A large population-based cohort study showed that long-term treatment of carvedilol is associated with a reduced risk of upper digestive tract and lung cancer and may be a potential drug to prevent these cancers [31]. Succinobucol-loaded nanoparticles can greatly inhibit lung metastasis of breast cancer [32]. Resveratrol alleviates the deterioration of a variety of human cancers, including gynecological cancer, kidney cancer, liver cancer, bladder cancer, prostate cancer, lung cancer, thyroid cancer, esophageal cancer, gastric cancer, colorectal cancer, and ovarian cancer. However, the potential therapeutic target and mechanism are not clear [33]. Some studies have found that CLEC14A methylation was associated with its expression and progression of lung adenocarcinoma and is a therapeutic target for solid tumors [34, 35]. Herein, we predicted that resveratrol can target methylation-driven gene CLEC14A and that the two were stably and tightly bound to each other through the formation of hydrogen bonds and hydrophobic interactions.

Finally, a risk score model composed of two methylation-driven genes was constructed through further analysis on 96 DMDGs. The progress of ELFN1-AS1 driving a variety of cancers, but whether its expression was affected by methylation was still unknown. A study has shown that ELFN1-AS1 drives progression of many cancers [36–38], but whether its expression is affected by methylation remains unknown. ZNF132 has been reported to be downregulated due to abnormally hypermethylation in prostate cancer [39] and lung cancer [40], and this is related to the prognosis of cancer. Herein, low expression of ZNF132 driven by DNA hypomethylation was also found to be significantly related to the poor prognosis of EC, and the signature composed of DMDGs showed a high accuracy in predicting the prognosis of EC. Using Sangerbox (http://vip.sangerbox.com), we also evaluated the prognostic relationship between ELFN1-AS1 and ZNF132 in other tumors. We filtered samples with a follow-up time of shorter than 30 days. It can be observed that ELFN1-AS1 is also significantly correlated with the poor prognosis of renal tumors, uveal melanoma, and liver cancer (Supplementary figure 2A), and ZNF132 is significantly correlated with the poor prognosis of leukemia and glioma (Supplementary figure 2B). It is a promising approach to improving diagnosis, prognosis, and treatment strategy based on methylation-driven genes, but more comprehensive researches are needed in the future for further verifying our results.

## Figures and Tables

**Table 1:** The grouping criteria of DMDGs.

Groups	Methylation cut-off	Expression cut-off
HypoUp	FDR < 0.01 and delta *β* value < -0.3	FDR < 0.01 and log2FC > 1
HypoDown	FDR < 0.01 and delta *β* value < -0.3	FDR < 0.01 and log2FC < −1
HyperUp	FDR < 0.01 and delta *β* value > 0.3	FDR < 0.01 and log2FC > 1
HyperDown	FDR < 0.01 and delta *β* value > 0.3	FDR < 0.01 and log2FC < −1

**Table 2:** 13 DMDGs and compounds that can interact with them.

Gene	GeneType	DrugCount	Drug example
BST1	HyperDown	5	Etheno-NAD, nicotinamide, adenosine 5′-[gamma-thio] triphosphate
CDO1	HyperDown	2	Cysteine, NADH
CLEC14A	HyperDown	1	Resveratrol
COX7A1	HyperDown	2	Cholic acid, N-formylmethionine
DDR2	HyperDown	2	Regorafenib, fostamatinib
FCER1A	HypoDown	3	Omalizumab, benzylpenicilloyl polylysine, TNX-901
GSTM5	HyperDown	2	Glutathione, glutathione disulfide
HBB	HypoDown	19	Iron dextran, zinc, 4-carboxycinnamic acid
RIC3	HyperDown	1	Theophylline
SERPINB5	HypoUp	1	S,S-(2-Hydroxyethyl)thiocysteine
SPARC	HyperDown	3	Calcium citrate, calcium phosphate, calcium phosphate dihydrate
VCAM1	HyperDown	4	Ethanol, carvedilol, succinobucol
XDH	HypoUp	9	Allopurinol, levocarnitine, topiroxostat

## Data Availability

All data generated or analyzed during this study are included in this article.

## Supplementary Materials

Supplementary 1 Supplementary Figure 1: correlation between ELFN1-AS1, ZNF132, and immune microenvironment infiltration.

Supplementary 2 Supplementary Figure 2: prognostic correlation of ELFN1-AS1 and ZNF132 in pan-cancer. (A) Prognostic significance of ELFN1-AS1 in pan-cancer. (B) Prognostic significance of ZNF132 in pan-cancer.
