# Supplementary figures and images for: Comprehensive Analysis of DNA Methylation and Transcriptome to Identify PD-1-Negative Prognostic Methylated Signature in Endometrial Carcinoma
Source: Dis Markers. 2022 May 18;2022:3085289. doi: 10.1155/2022/3085289 (PMC9133896; doi:10.1155/2022/3085289)

# ELFN1-AS1

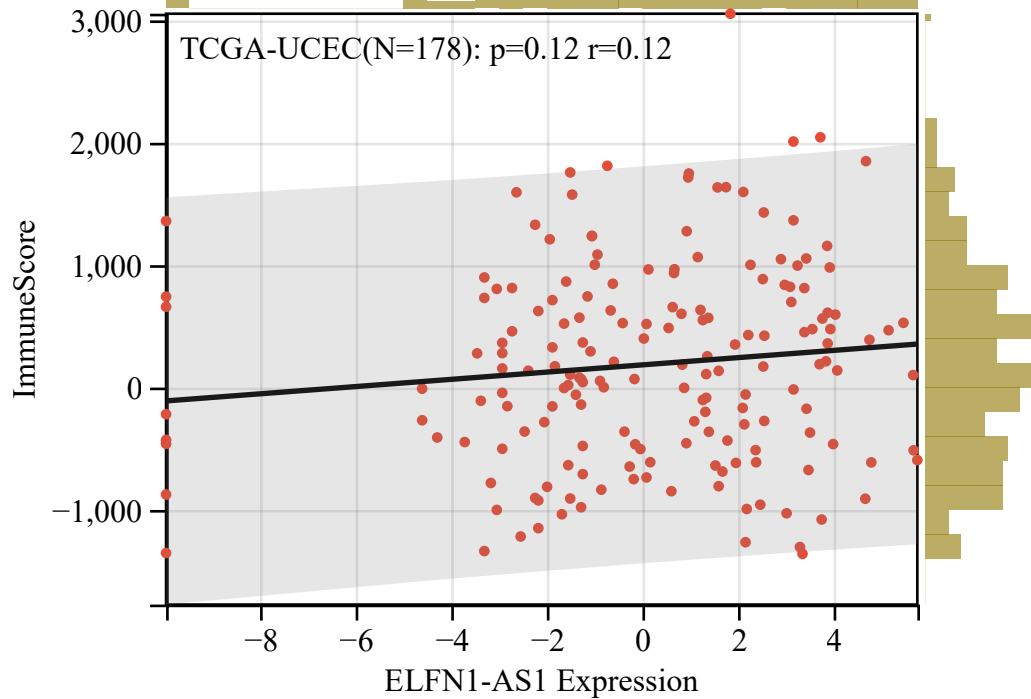

# ZNF132

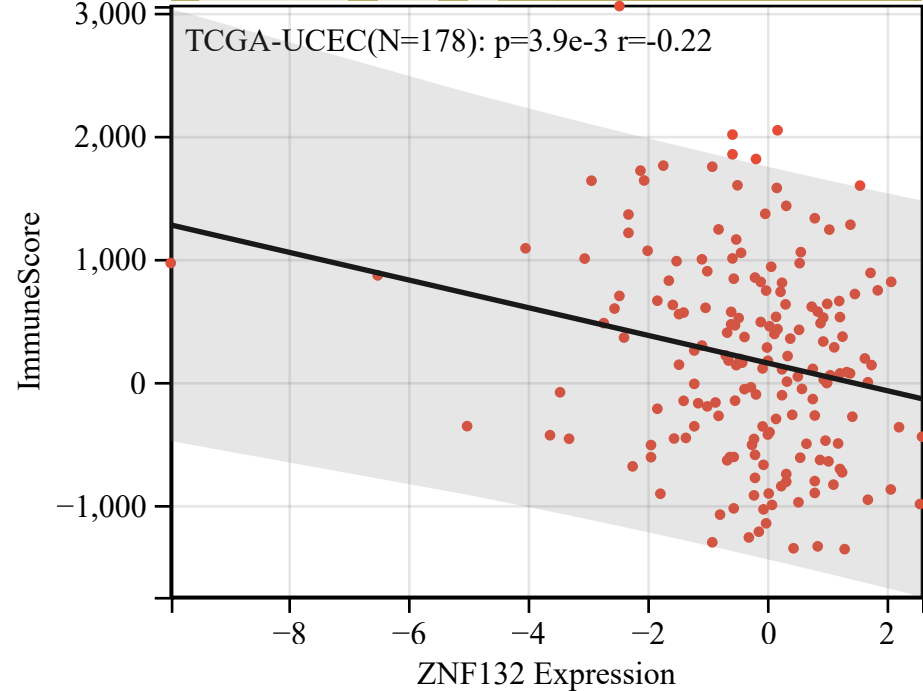

Supplement: Supplementary 1 — Supplementary Figure 1: correlation between ELFN1-AS1, ZNF132, and immune microenvironment infiltration. [file 3085289.f1.pdf]

A

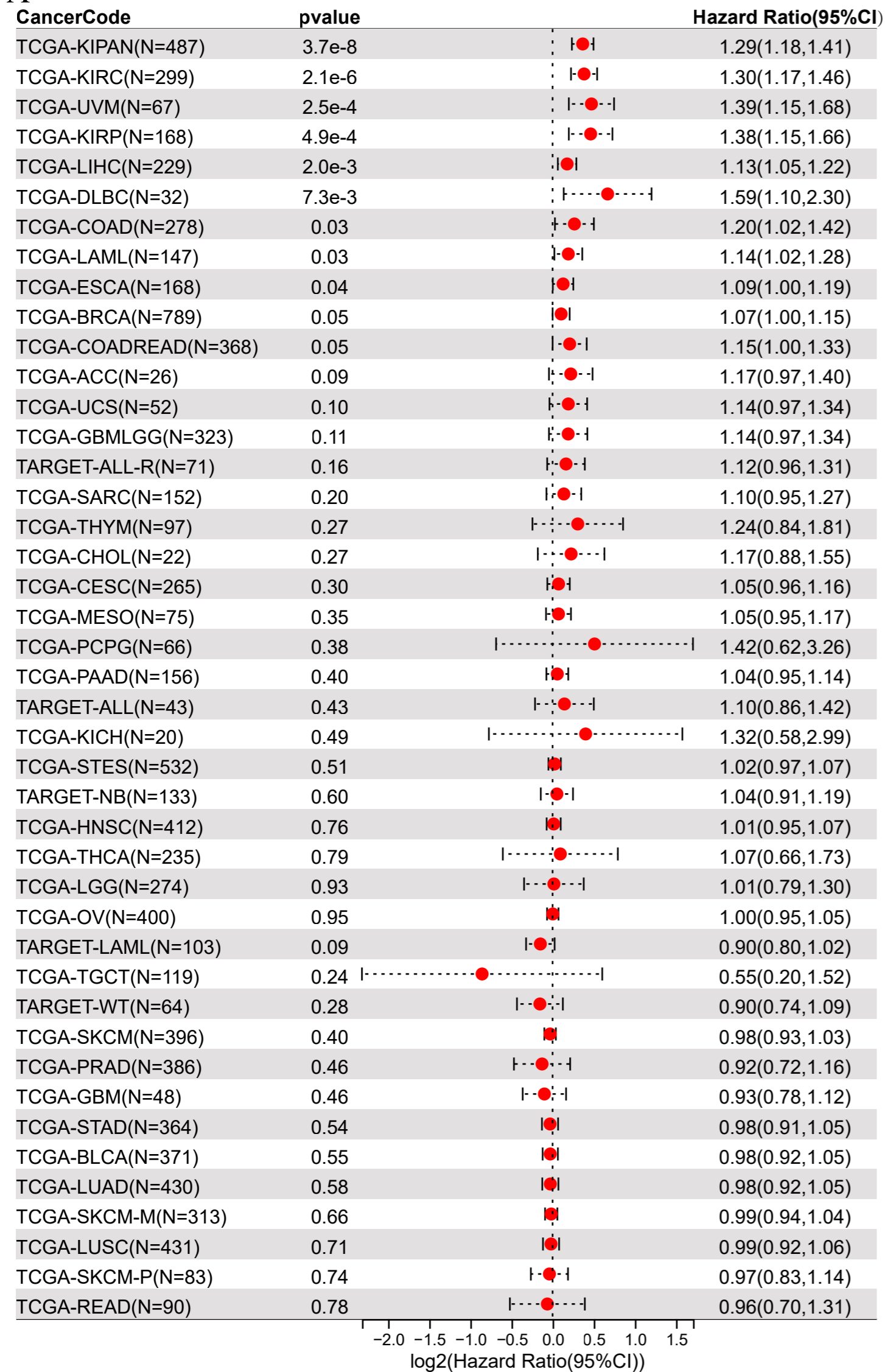

B

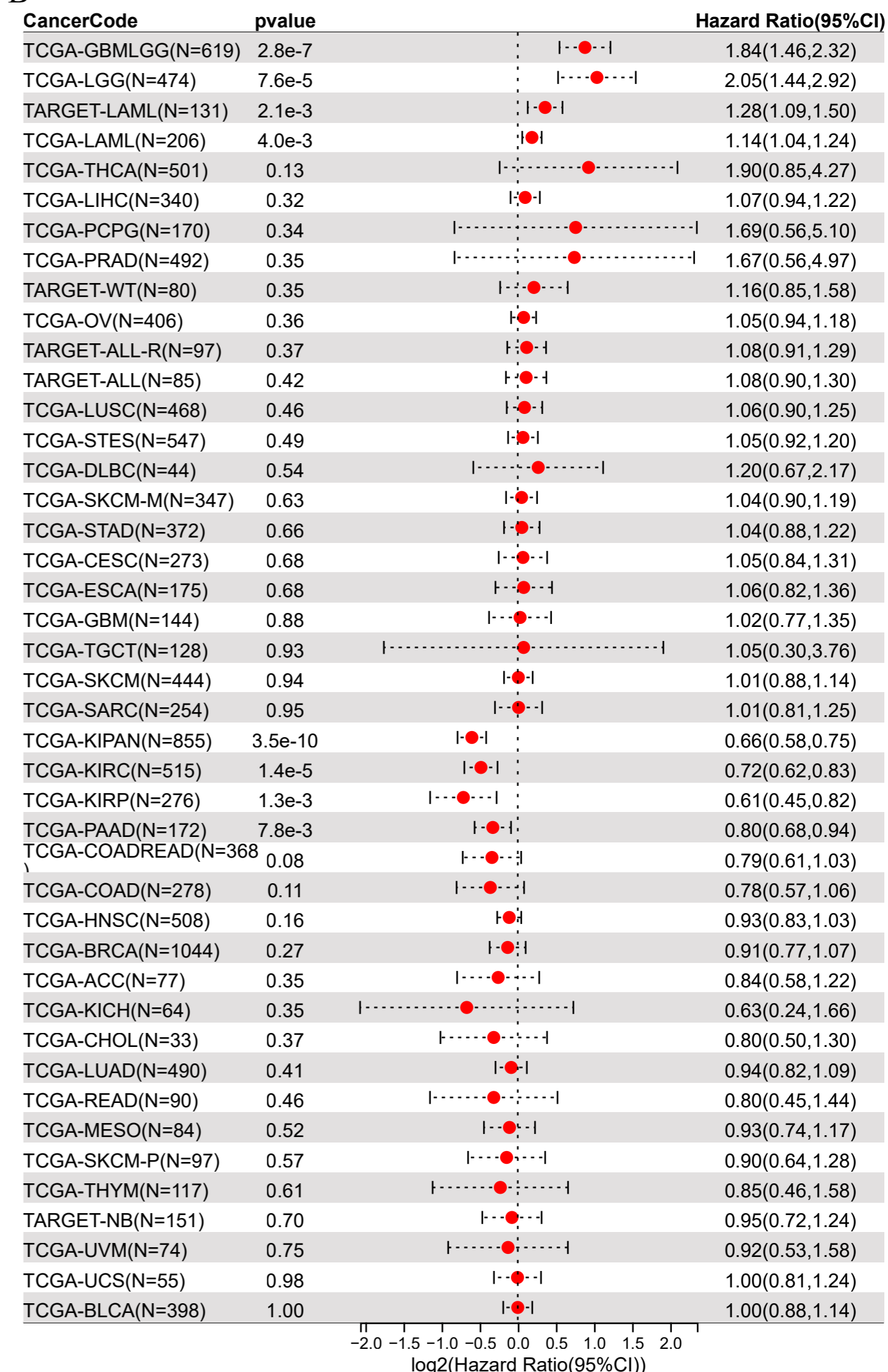

Supplement: Supplementary 2 — Supplementary Figure 2: prognostic correlation of ELFN1-AS1 and ZNF132 in pan-cancer. (A) Prognostic significance of ELFN1-AS1 in pan-cancer. (B) Prognostic significance of ZNF132 in pan-cancer. [file 3085289.f2.pdf]
